# Role of some serum biomarkers in the early detection of diabetic cardiomyopathy

**DOI:** 10.2144/fsoa-2020-0184

**Published:** 2021-02-04

**Authors:** Amany H Abdelrahman, Iman I Salama, Somaia I Salama, Dalia M Elmosalami, Mona H Ibrahim, Eman M Hassan, Mark O Dimitry, Zahraa I Aboafya, Mohammad Gouda Mohammad, Mohamed Amin

**Affiliations:** 1 Department of Clinical & Chemical Pathology, National Research Centre, Cairo, Egypt; 2 Department of Community Medicine Research, National Research Centre, Cairo 12622, Egypt; 3 Department of Internal Medicine, National Research Centre, Cairo, Egypt; 4 Department of Cardiovascular Medicine, Faculty of Medicine, Zagazig University, Zagazig, Egypt

**Keywords:** AGEs, diabetic cardiomyopathy, diastolic dysfunction, IGFBP-7, IL-6, insulin, TNF-α

## Abstract

**Aim:**

To assess the role of serum biomarkers in early prediction of diabetic cardiomyopathy.

**Materials and methods:**

The participants were three groups of Type 2 diabetes mellitus (DM) patients having diastolic dysfunction (DM-DD), systolic dysfunction (DM-SD) and normal echocardiography (DM-N) with two control groups: non-DM diastolic dysfunction patients (DD) and healthy controls. AGEs, TNF-α, IL-6, IGFBP-7, creatinine and insulin were assessed.

**Results:**

TNF-α, AGEs, creatinine and insulin panel had area under the curve (AUC) of 0.913 in distinguishing DM-DD from DM-N (78.7% sensitivity and 100% specificity). IL-6 and AGEs panel had AUC 0.795 for differentiating DM-SD from DM-DD (90.6% sensitivity). IL-6, TNF-α and AGEs panel had AUC 0.924 for differentiating diabetic cardiomyopathy from DM-N (85% sensitivity and specificity).

**Conclusion:**

A panel of AGEs, IL-6, TNF-α, insulin and creatinine might be used for early detection of DM-DD among T2DM patients.

## Lay abstract

Diabetic cardiomyopathy is a disorder of the heart muscle among diabetic people. The early stage of diabetic cardiomyopathy is reversible, while later stages progress to heart failure. We were able to identify a panel of serum biomarkers that can be used for detection of the reversible stage of diabetic cardiomyopathy with 90% sensitivity.

## Graphical abstract

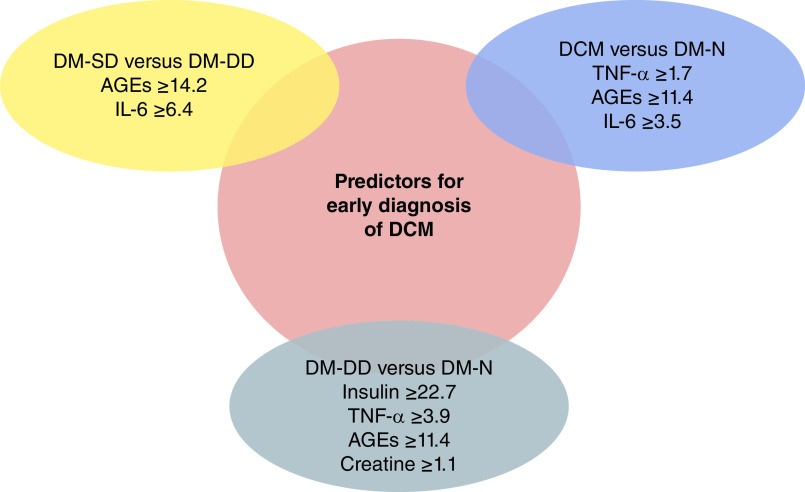

Diabetic cardiomyopathy (DCM) is a potential complication of diabetes mellitus (DM) [1]. Cardiac involvement in patients with DM may occur relatively early in the course of disease, impairing left ventricular relaxation (diastolic dysfunction) and later on can affect ventricular contraction (systolic dysfunction) [2]. As diabetes usually co-exists with other diseases as ischemic heart disease and hypertension, Lee *et al.* [3] suggested to define DCM as cardiac disorders that can be attributed to diabetes and could not be explained by other cardiovascular or noncardiovascular diseases. Diastolic dysfunction (DD) may be considered as the first functional abnormality in DCM and can be detected in 40–60% of asymptomatic diabetic patients using echocardiography [4]. DCM usually begins with myocardial fibrosis, dysfunctional remodeling and associated DD, followed by systolic dysfunction (SD), ending by heart failure (HF) [5].

Hyperglycemia, hyperinsulinemia and insulin resistance may lead to cardiac insulin resistance and metabolic disturbances that aggravate oxidative stress, mitochondria dysfunction and increase in advanced glycation end-products (AGEs). These abnormalities increase cardiac hypertrophy, stiffness and fibrosis resulting in DCM [6]. TNF-α and IL-6 are multifunctional cytokines detected in DCM. They are implicated in the progression of HF through induction of cardiac cell apoptosis via increasing oxidative stress and ligand-receptor cell death signals [7]. IGFBP-7 is recognized as a biomarker for DD accompanied with myocytes fibrosis, cardiac hypertrophy and vascular remodeling [8].

In the later stages of DCM, it progresses from DD to apparent stage of HF with conserved ejection fraction, which has no confirmed successful treatment [1]. This emphasizes the importance of detecting biomarkers that can enhance diagnosis of DCM before the occurrence of permanent complications. The current study aimed at evaluating serum biomarkers TNF-α, IL-6, IGFBP-7, AGEs, insulin and creatinine alone or in combination with each other to predict early-stage of DCM.

## Materials & methods

The current work is a case–control study. The studied participants were aged from 42 to 69 years and were recruited from Zagazig University Hospital and National Research Centre (NRC). Using echocardiography, the studied T2DM patients were classified into three groups: 47 patients with DM-DD, 32 patients with systolic dysfunction (DM-SD) and 34 patients with normal cardiac function (DM-N). Another two groups: 33 non-diabetics with DD and 31 non-diabetic with normal echocardiography subjects, were included as control groups. They were recruited from NRC employees of comparable age and sex to the T2DM patients. Subjects were excluded if they had any evidence of antecedent myocardial infarction, known congenital or valvular heart disease, malignancy, renal failure, significant psychiatric illness, history of taking an anti-oxidative-stress drug such as α-lipoic acid, vitamin C or E, within the past month.

Interviews were carried out with all the studied participants to collect data about their demography and medical history. History of diabetes was taken from T2DM patients including age of onset of diabetes, number and frequency of hyperglycemic or hypoglycemic comas and type of treatment taken for diabetes (insulin or oral hypoglycemic drug). All the studied subjects were asked about cardiovascular manifestation with emphasis on the presence of dyspnea, tachycardia, hypertension and lower limb edema. All the studied individuals were subjected to thorough clinical examination and anthropometric assessment for height and weight in order to estimate the BMI as a measure of obesity. Systolic and diastolic blood pressures was measured to the nearest even digit from the right arm of the seated participant. Hypertension is defined as recurrent elevation of blood pressure exceeding 140/90 mmHg or current use of antihypertensive medications.

### Laboratory analysis

A barcode, which resembles a unique identification number, was assigned for each subject. Venous blood sample of 10 ml was aseptically withdrawn from each participant. The sample was divided into three tubes. About 2 ml of blood sample were put on ethylonethylenediamine tetra-acetic acid, dipotassium salt (K2-EDTA) in vacutainer tube (final concentration of 1.5 mg/ml) for measurement of glycated hemoglobin (HbA1c). For chemical lab analysis, 4 ml of blood sample were put in a plain vacutainer tube for measurement of fasting blood sugar, cholesterol, triglyceride, high density lipoprotein cholesterol, low density lipoprotein cholesterol and creatinine by enzymatic colorimetric method using Erba XL-300. Last 4 ml were put in a plain vacutainer tube for measurement of level of AGEs, inflammatory cytokines: TNF-α and IL-6, pro-fibrotic markers: IGFBP-7 and fasting insulin using ELISA. AGEs, IGFBP-7 and insulin levels were assessed using commercial kits supplied by Bioassay Technology Laboratory (Cat No: E0003Hu, E3857 Hu and E0010Hu, respectively). TNF-α was assessed using commercial kit supplied by Affymetrix eBioscience (Cat No: BMS233/4TEN). IL-6 was assessed using commercial kit supplied by Invitrogen (Cat No: EH2IL6). ELISAs were conducted according to the manufacturer’s protocol.

### Echocardiography

For all the studied participants, 2D Doppler echocardiogram with color flow imaging was carried out and measurements were obtained according to American Society of Echocardiography guidelines. These measurements included posterior wall thickness (PWT), ejection fraction (EF%), septal wall thickness, left ventricular internal dimension (LVID) and left atrial size. Relative wall thickness was calculated according to formula: (2 × PWTd)/LVIDd [9]. Mitral valve inflow Doppler was recorded, including E and A waves. Tissue Doppler was used for E’ and A'. E/e' was calculated as LV filling pressure. In patients with a normal left ventricular ejection fraction (LVEF), DD was based on the assessment of four variables: septal e' <7 cm/sec or lateral e' <10 cm/sec, average E/e' >14, LA volume index >34 ml/m^2^ and peak TR velocity >2.8 m/sec [10]. The measurement of left ventricle (LV) dimensions, EF% and fractional shortening % were assessed to evaluate the systolic functions using M-mode tracing in parasternal long axis and short axis views. EF% is the percent change of left ventricular chamber volumes between diastole and systole from apical four and two chamber views using biplane Simpson's rule, EF >55% indicated a normal systolic function, 50–55% a borderline systolic functions and <50% a reduced systolic functions [11].

### Statistical analysis

Data entry and analysis were done using SPSS version 18.0 for Windows from SPSS, Inc. (IL, USA). Chi square was done for qualitative data that presented by numbers and percentages. Continuous data were expressed as mean and standard deviation. Student t-test was used to compare between two means and ANOVA to compare between more than two means. When data are not normally distributed, nonparametric Mann–Whitney and the Kruskal–Wallis tests were used for comparing two or more independent samples. Receiver-operating characteristic (ROC) analysis was carried out for obtaining the area under the curve (AUC) and the corresponding 95% CI. The maximum diagnostic discrimination cutoff point was evaluated, corresponding to the highest Youden index for each biomarker. Logistic regression analysis was used to identify the significant predicting biomarkers (using the maximum diagnostic discrimination cutoff points) to differentiate between patients with DM-DD from those with DM-SD or DM-N and patients with DCM from DM-N. For each logistic model, the predicted probability for the significant biomarkers panel together were calculated. Panel of the significant biomarkers was validated using the algorithm (see Box 1 for algorithms). ROC curve analyses were done to assess the AUC for each significant biomarkers and effect of using a panel of these biomarkers together. The sensitivity, specificity, positive predictive values (PPV) and negative predictive values (NPV) were calculated for the identified cutoff’ points for each biomarker. The p-value is statistically significant when it is <0.05 and considered statistically highly significant if it is <0.01.

Box 1.  **Algorithms of the biomarker panels for predicting DCM**

**1-Probability of having DM-DD distinguish from DM-N**

EXP(2.625* insulin [uIU/ml] + 1.593 * TNF-α [pg/ml] + 1.934* AGEs [ng/ml] + 2.066 [creatinine mg/dl] - 2.142)/(1 + EXP(2.625* insulin [uIU/ml] + 1.593 * TNF-α [pg/ml] + 1.934* AGEs [ng/ml] + 2.066 [creatinine mg/dl] - 2.142)

**2-Probability of having DM-SD distinguish from DM-DD**

EXP(2.189* IL-6 [pg/ml] + 1.188* AGEs [ng/ml] - 2.507)/(1 + EXP(2.189* IL-6 [pg/ml] + 1.188* AGEs [ng/ml] - 2.507)

**3-Probability of having diabetic cardiomyopathy distinguish from DM-N**

EXP(1.947* IL-6 [pg/ml] + 2.217* AGEs [ng/ml] + 1.783* TNF-α [pg/ml] - 1.667) / (1 + EXP(1.947* IL-6 [pg/ml] + 2.217* AGEs [ng/ml] + 1.783* TNF-α [pg/ml] - 1.667)

AGE: Advanced glycation end-product; DD: Diastolic dysfunction; DM: Diabetes mellitus; N: normal echocardiography; SD: Systolic dysfunction.

## Results

Table 1 shows that there was no significant difference between the studied groups as regards age, sex, smoking and BMI, p > 0.05. The percent of individuals with hypertension was significantly different among the studied groups with the highest percent among DM-DD; p < 0.001. Table 2 shows laboratory analysis of the studied biomarkers among T2DM patients and the two control groups. There was a significant difference between the studied groups regarding different biomarkers; p < 0.001. Both DM-DD and DM-SD patients had significantly elevated mean serum level of TNF-α, IL-6, insulin, AGEs and creatinine compared with DM-N patients and the two control groups; p < 0.001. Among the two control groups, there was no significant difference between DD group and normal echocardiography group in all biomarkers except for cholesterol, triglycerides and high density lipoprotein cholesterol with p < 0.001.

**Table 1.** Clinical characteristics of the studied Type 2 diabetes mellitus patients and the two control groups.

Variables	DM-N n = 34 n (%)	DM-DD n = 47 n (%)	DM-SD n = 32 n (%)	DD n = 33 n (%)	Controls n = 31 n (%)	p-value
Age (mean ± SD)	54.7 ± 4.1	56.1 ± 5.7	55.7 ± 8.1	53.7 ± 5.4	55.9 ± 3.6	0.125
Gender						
Males	16 (47.1)	21 (44.7)	15 (46.9)	15 (45.5)	15 (48.4)	0.173
Females	18 (52.9)	26 (55.3)	17 (53.1)	18 (54.5)	16 (51.6)	
Smoking						
Nonsmokers	30 (88.2)	42 (89.4)	18 (56.3)	26 (78.8)	23 (74.2)	0.23
Smoker/exsmokers	4 (11.8)	5 (10.6)	14 (43.8)	7 (21.2)	9 (25.8)	
BMI	34.6 ± 5.9	33.5 ± 7.2	29.7 ± 5.1	32.1 ± 4.8	31.7 ± 8.4	0.085
History of hypertension						
Yes	16 (47.1)	34 (72.3)	12 (37.5)	16 (48.5)	5 (16.1)	<0.001^†^
No	18 (52.9)	13 (27.7)	20 (62.5)	17 (51.5)	26 (83.9)	

† p < 0.01 is considered highly significant.

AGE: Advanced glycation end-product; DD: Diastolic dysfunction; DM: Diabetes mellitus; N: Normal echocardiography; SD: Systolic dysfunction.

**Table 2.** Laboratory analysis of the laboratory biomarkers among Type 2 diabetes mellitus patients and the two control groups.

Biomarkers	DM-N n = 34 Mean ± SD	DM-DD n = 47 Mean ± SD	DM-SD n = 32 Mean ± SD	DD n = 33 Mean ± SD	Controls n = 31 Mean ± SD	p-value
TNF-α (pg/ml)	1.8 ± 2.1	5.2 ± 3.2^¶^^#^	5.8 ± 3.6^‡‡^^§§^	2.1 ± 2.4^¶¶^	1.4 ± 0.5	<0.001^‡^
IL-6 (pg/ml)	2.0 ± 0.7	18.3 ± 26.2^¶^^#^	24.1 ± 17.9^‡‡^^§§^	2.2 ± 1.6^¶¶^	1.5 ± 0.3	<0.001^‡^
Insulin (uIU/ml)	15.9 ± 8.7	54.5 ± 57.2^¶^^#^	56.9 ± 57.3^‡‡^^§§^	14.8 ± 8.6^¶¶^	6.7 ± 1.4	<0.001^‡^
AGEs (ng/ml)	9.1 ± 1.3	12.9 ± 5.4^¶^^#^^††^	15.6 ± 4.7^‡‡^^§§^	7.7 ± 2.5^¶¶^	7.8 ± 1.0	<0.001^‡^
IGFBP-7 (ng/ml)	3.5 ± 1.1	4.6 ± 2.4^#^^††^	6.8 ± 6.2^‡‡^^§§^	2.7 ± 0.9^¶¶^	2.8 ± 0.8	<0.001^‡^
Creatinine (mg/dl)	0.8 ± 0.2	1.1 ± 0.4^¶^^#^^††^	1.3 ± 0.5^‡‡^^§§^	0.8 ± 0.2^¶¶^	0.8 ± 0.1	<0.001^‡^
Cholesterol (mg/dl)	195.2 ± 49.5	168.9 ± 44.6^¶^	149.9 ± 42.3^‡‡^^††^	211.6 ± 42.9^¶¶^^##^	182.2 ± 30.9	<0.001^‡^
Triglyceride (mg/dl)	121.1 ± 59.8	145.7 ± 57.1	126.3 ± 45.8	143.2 ± 63.7^##^	90.2 ± 24.2	<0.001^‡^
HDL-C (mg/dl)	53.9 ± 16.5^§^	36.0 ± 8.9^¶^	31.9 ± 7.1^‡‡^^§§^	45.5 ± 7.1^¶¶^^##^	40.5 ± 14.4	<0.001^‡^
LDL-C (mg/dl)	117.1 ± 41.9	103.5 ± 36.8	94.1 ± 37.6^‡‡^	121.5 ± 35.6^¶¶^	106.5 ± 14.5	0.01^†^
FBS (mg/dl)	161.8 ± 64.1^§^	217.1 ± 84.7^¶^^#^^††^	187.0 ± 72.0^§§^	95.6 ± 12.3^¶¶^	89.4 ± 12.9	<0.001^‡^
HbA1c%	7.6 ± 1.7^§^	7.8 ± 1.6^#^	8.0 ± 1.9^§§^	5.1 ± 0.6^¶¶^	5.0 ± 0.3	<0.001^‡^

† p < 0.05 is considered significant.

‡ p < 0.01 is considered highly significant.

§ Significant difference between DM-N and controls.

¶ Significant difference between DM-DD and DM-N.

# Significant difference between DM-DD and Controls.

†† Significant difference between DM-DD and DM-SD.

‡‡ Significant difference between DM-SD and DM-N.

§§ Significant difference between DM-SD and controls.

¶¶ Significant difference between DD and DM-SD, DM-DD.

## Significant difference between DD and controls.

AGE: Advanced glycation end-product; DD: Diastolic dysfunction; DM: Diabetes mellitus; HDL-C: High density lipoprotein cholesterol; LDL-C: Low density lipoprotein cholesterol; N: Normal echocardiography; SD: Systolic dysfunction.

Logistic regression analysis revealed that the level of insulin ≥22.7, TNF-α ≥3.9, AGEs ≥11.6, creatinine ≥1.1 were the significant predicting factors for DM-DD from DM-N with adjusted odds ratio (AOR) 13.8, 4.9, 6.9 and 7.8, respectively; p < 0.05. Meanwhile, AGEs ≥14.2 and IL-6 ≥6.4 were the significant predicting factors for DM-SD from DM-DD with AOR 3.2 and 8.9, respectively; p < 0.05. The significant predicting biomarkers for DCM from DM-N were TNF-α ≥1.7, AGEs ≥11.4 and IL-6 ≥3.5 with AOR 5.9, 9.1 and 7.0, respectively; p < 0.05 (Table 3).

**Table 3.** Logistic regression analysis for predicting the risk of different types of diabetic cardiomyopathy.

Variable	Logistic co-efficient	Adjusted odds ratio	95% CI		p-value
			Lower	Upper	
DM-DD vs DM-N					
Insulin ≥22.7	2.625	13.807	3.160	60.333	<0.001^‡^
TNF-α ≥3.9	1.593	4.918	1.173	20.630	0.029^†^
AGE ≥11.4	1.934	6.914	1.026	46.595	0.047^†^
Creatinine ≥1.1	2.066	7.895	1.247	49.979	0.028^†^
Constant	-2.142	0.117			<0.001^‡^
DM-SD vs DM-DD					
AGE ≥14.2	1.188	3.281	1.135	9.485	0.028^†^
IL6 ≥6.4	2.189	8.925	2.298	34.661	0.002^‡^
Constant	-2.507	0.082			<0.001^‡^
DCM vs DM-N					
TNF-α ≥1.7	1.783	5.945	1.666	24.749	0.014^†^
AGE ≥11.4	2.217	9.177	1.428	50.564	0.011^†^
IL6 ≥3.5	1.947	7.009	1.540	31.901	0.012^†^
Constant	-1.667	.189			<0.001^‡^

† p < 0.05 is considered significant.

‡ p < 0.01 is considered highly significant.

AGE: Advanced glycation end-product; DCM: Diabetic cardiomyopathy; DD: Diastolic dysfunction; DM: Diabetes mellitus; N: Normal echocardiography; SD: Systolic dysfunction.

**Figure 1.**
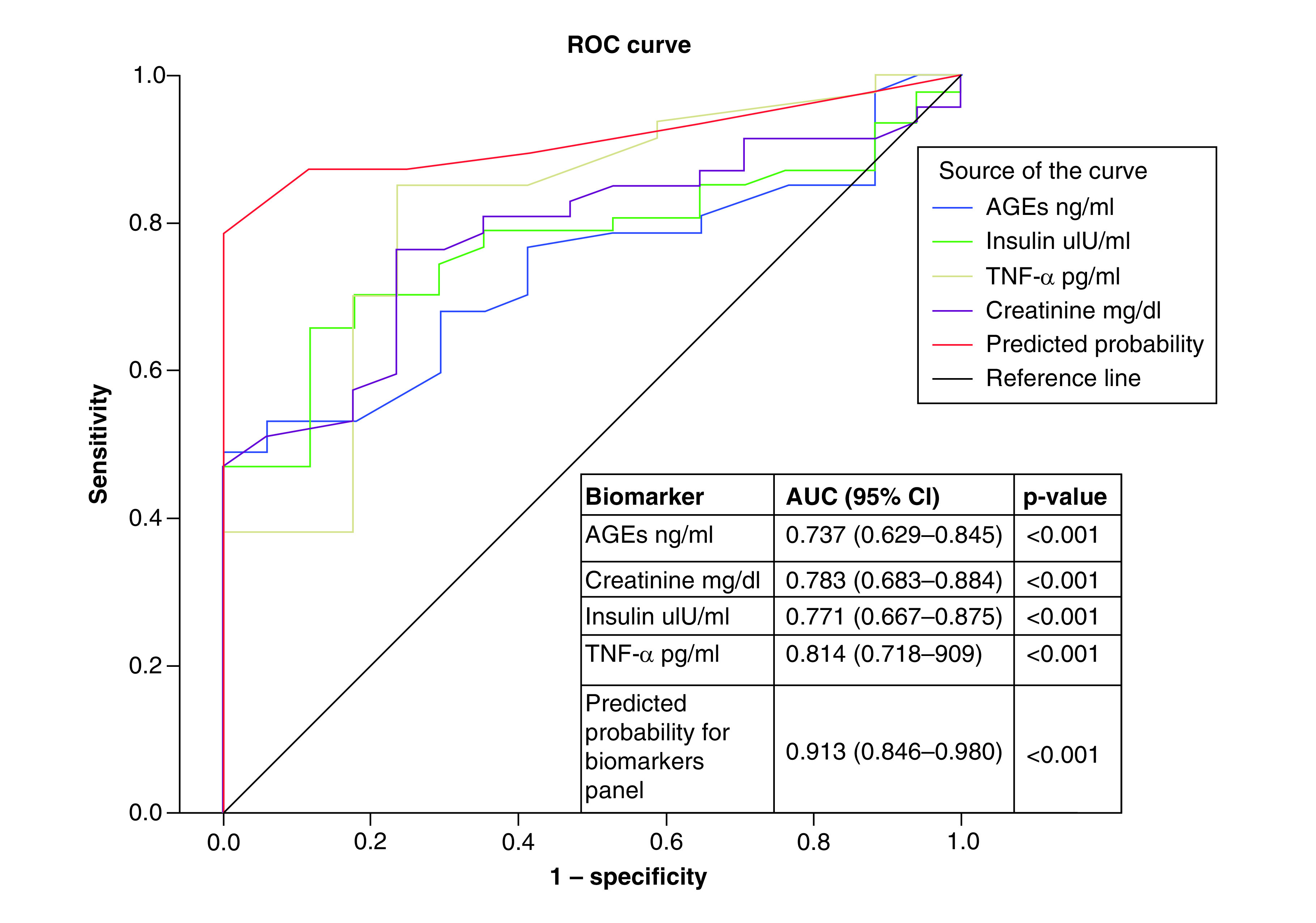
ROC curve and AUC for laboratory biomarkers for prediction of Type 2 diabetes mellitus patients with diastolic dysfunction versus Type 2 diabetes mellitus normal cardiac function. AGE: Advanced glycation end-product; AUC: Area under the curve; ROC: Receiver operating characteristic.

**Figure 2.**
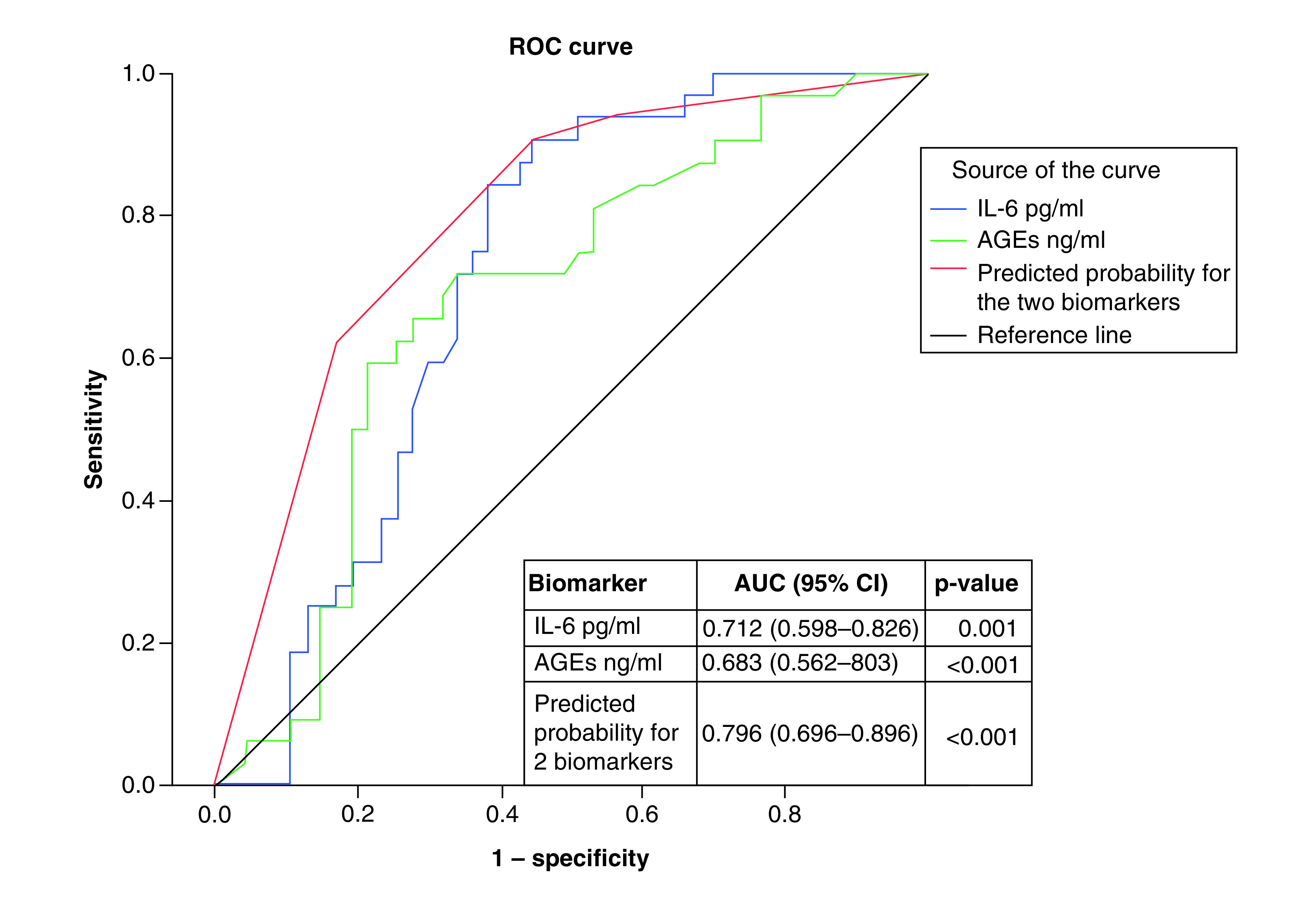
ROC curve and AUC for laboratory biomarkers for prediction of Type 2 diabetes mellitus patients with systolic dysfunction versus diastolic dysfunction. AGE: Advanced glycation end-product; AUC: Area under the curve; ROC: Receiver operating characteristic.

**Figure 3.**
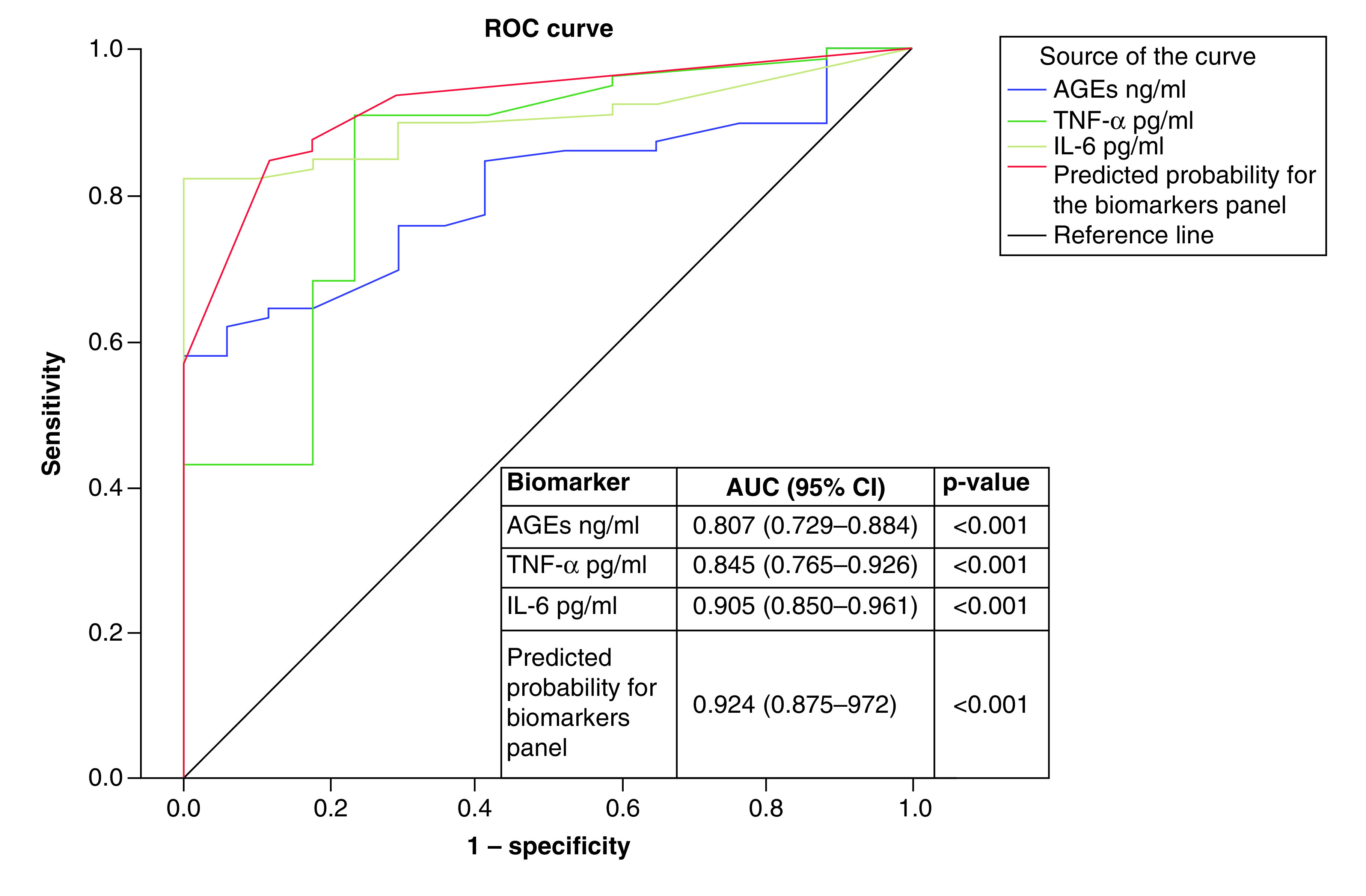
ROC curve and AUC for laboratory biomarkers for prediction of Type 2 diabetes mellitus patients with diabetic cardiomyopathy versus Type 2 diabetes mellitus normal cardiac function. AGE: Advanced glycation end-product; AUC: Area under the curve; ROC: Receiver operating characteristic.

Figures 1–3 show the results of ROC curve analysis and AUC of the different studied biomarkers for prediction of DM-DD, DM-SD and DCM. Figure 1 shows T2DM patients with DD versus DM-N, where AUC for AGEs, creatinine, insulin and TNF-α were 0.737, 0.783, 0.771 and 0.814, respectively; p < 0.001. A panel of these biomarkers together had excellent performance in detecting DM-DD from to DM-N with an AUC of 0.913 (p < 0.001). For prediction of DM-SD versus DM-DD, the AUC for IL-6, AGEs and a combination panel of these two biomarkers were 0.712, 0.683 and 0.796, respectively; p < 0.001 (Figure 2). Prediction of DCM (diastolic and systolic) versus DM-N is presented in Figure 3, where AUC of AGEs, TNF-α and IL-6 were 0.807, 0.845 and 0.905, respectively; p < 0.001. A panel of these biomarkers together had excellent performance in detecting DCM from DM-N with an AUC of 0.924 (p < 0.001).

Table 4 shows sensitivity, specificity, PPV and NPV of different studied biomarkers at the chosen cutoff points to differentiate between DM-DD from DM-N and DM-SD patients and between DCM and DM-N patients. The biomarkers TNF-α ≥3.9, insulin ≥22.7, AGEs ≥11.6 and creatinine ≥1.07 differentiated DM-DD from DM-N with 82.3–94.1% specificity, where TNF-α ≥3.9 showed the highest sensitivity. A panel of these biomarkers increased the specificity to 100%. To differentiate DM-SD from DM-DD, the biomarker IL-6 ≥6.4 demonstrated 90.6% sensitivity. Serum level of TNF-α ≥1.7, IL-6 ≥3.5 and AGEs ≥11.4 could differentiated DCM from DM-N with 58.2–89.9%, where TNF-α ≥1.7 showed highest sensitivity. A panel of these biomarkers increased the specificity and sensitivity to 88.2 and 84.8%, respectively.

**Table 4.** Biomarkers cutoff levels with sensitivity, specificity, positive and negative predictive values for differentiation between diabetic cardiomyopathy and normal cardiac function among Type 2 diabetes mellitus patients.

Biomarker cutoff level	DM-DD n = 47 n (%)	DM-N n = 34 n (%)	p-value^†^	Youden index	Sensitivity	Specificity	PPV	NPV
TNF-α ≥3.9	33 (70.2)	6 (17.6)	<0.001	0.526	70.2%	82.4%	84.6%	66.7%
Insulin ≥22.7	31 (66.0)	4 (11.8)	<0.001	0.542	66.0%	88.2%	88.6%	65.2%
AGEs ≥11.6	23 (48.9)	2 (5.9)	<0.001	0.489	48.9%	94.1%	92.0%	57.1%
Creatinine ≥1.1	24 (51.1)	2 (5.9)	<0.001	0.468	51.1%	94.1%	92.3%	58.2%
Panel of biomarkers (probability 0.709)	37 (78.7)	0 (0.0)	<0.001	0.787	78.7%	100.0%	100.0%	77.3%

**Biomarker cutoff level**	**DM-SD** **n = 32** **n (%)**	**DM-DD** **n = 47** **n (%)**	**p-value**^†^	**Youden index**	**Sensitivity**	**Specificity**	**PPV**	**NPV**
IL-6 ≥6.4	29 (90.6)	21 (44.7)	<0.001	0.459	90.6%	55.3%	58.0%	89.7%
AGEs ≥14.2	21 (65.6)	13 (27.7)	0.001	0.380	65.6%	72.3%	61.8%	75.6%
Panel of biomarkers (probability 0.316)	29 (90.6)	21 (44.7)	<0.001	0.459	90.6%	55.3%	58.0%	89.7%

**Biomarker cutoff level**	**DCM** **n = 79** **n (%)**	**DM-N** **n = 34** **n (%)**	**p-value**^†^	**Youden index**	**Sensitivity**	**Specificity**	**PPV**	**NPV**
TNF-α ≥1.7	71 (89.9)	8 (23.5)	<0.001	0.663	89.9%	76.5%	89.9%	76.5%
IL-6 ≥3.5	65 (82.3)	4 (11.8)	<0.001	0.823	82.3%	88.2	94.2%	68.2%
AGEs ≥11.4	46 (58.2)	2 (5.9)	<0.001	0.582	58.2%	94.1%	95.8%	49.2%
Panel of biomarkers (probability 0.760)	67 (84.8)	4 (11.8)	<0.001	0.730	84.8%	88.2%	94.4%	71.4%

† p < 0.01 is considered highly significant.

AGEs: Advanced glycation end-products; DCM: Diabetic cardiomyopathy; DD: Diastolic dysfunction; DM: Diabetes mellitus; N: Normal echocardiography; NPV: Negative predictive value; PPV: Positive predictive value; SD: Systolic dysfunction.

## Discussion

DM and its associated complications constitute a global burden on individual health and economics [12]. Cardiovascular diseases are the principal cause of death among patients with DM [13]. The current study demonstrated that for distinguishing between DM-DD from DM-N, AUC for TNF-α, AGEs, creatinine and insulin, were found to be over 0.737; p < 0.01. A panel of these four biomarkers significantly increased AUC to 0.913 and specificity to 100%. Meanwhile, for differentiating DM-SD from DM-DD it was 0.712 for IL-6 and 0.683 for AGEs. A panel of these two biomarkers significantly increased AUC to 0.795 and increased sensitivity to 90.6%. For discrimination between DCM patients from DM-N, IL-6, TNF-α and AGEs had AUCs of 0.905, 0.845 and 0.807, respectively. A panel of these biomarkers significantly increased AUC to 0.924, increasing sensitivity to 84.8% and specificity to 88.2%. Therefore, ROC curve analysis strongly supports that the identified biomarkers were sensitive enough to detect the early onset of DCM.

The current work revealed that DM-DD and DM-SD patients had significantly elevated TNF-α, IL-6, insulin, AGEs and creatinine compared with DM-N and controls; p < 0.001. Logistic regression analysis revealed that cutoff level of insulin ≥22.7, TNF-α ≥3.9, AGEs ≥11.6, creatinine ≥1.1 were the significant predicting factors for DM-DD versus DM-N with AOR 13.8 (3.1–60.3), 4.9 (1.1–20.6), 6.9 (1.0–46.5) and 7.8 (1.2–49.9), respectively; p < 0.05. Meanwhile, AGEs ≥14.2 and IL-6 ≥6.4 were the significant predicting factors for DM-SD versus DM-DD with AOR 3.2 (1.1–9.4) and 8.9 (2.2–34.6), respectively; p < 0.05. The significant predicting biomarkers for DCM versus DM-N were TNF-α ≥1.7, AGEs ≥11.4 and IL-6 ≥3.5 with AOR 5.9 (1.6–24.7), 9.1 (1.4–50.5) and 7.0 (1.5–31.9), respectively; p < 0.05. A panel of the significant biomarkers was validated using an algorithm (see Box 1 for algorithms). Multivariate analyses by Haugen *et al.* [14] revealed that IL-6 was a significant risk factor for HF. In a large cohort study carried out by George *et al.* [15] among HF patients, appropriately half had IL-6 levels above the 95th percentile of normal values. They recommended further investigation into IL-6 as a potential therapeutic target for patients with HF. Shaver *et al.* [16] and Dinh *et al.* [17] demonstrated elevated levels of IL-6 and TNF-α among DM-DD patients compared with controls. Additionally, several reports have illustrated increased expression and release of inflammatory cytokines such as TNF-α, IL-6 in the plasma and within the failing myocardium in direct proportion to deterioration of cardiac functional class and performance [18]. Previous studies have indicated the role of TNF-α and IL-6 in cardiac remodeling, fibrosis, cardiomyocyte apoptosis and ischemic heart disease [19]. Haugen *et al.* [20] reported that in heart biopsies of rats having diastolic dysfunction, there was an increase of mRNA levels for IL-6, with upregulation of IL-6. This might indicate active pro-inflammatory process as an underlying mechanism during the early stage when cardiac hypertrophy associated with diastolic dysfunction occurs.

In the current study, AGEs levels were significantly higher among patients with DM-SD and DM-DD compared with DM-N and controls; p < 0.001. The mean serum AGEs level was significantly higher among DM-SD (15.6 ± 4.7) compared with DM-DD (12.9 ± 5.4). Moreover, the AGEs cutoff for predicting DM-SD from DM-DD (≥14.2) was higher than that needed to predict DM-DD from DM-N (≥11.6). Hyperglycemia facilitates the reaction of glucose with collagen to form the AGEs [21]. Studies carried out on human or animal myocardium revealed that cardiac accumulation of AGEs in DM patients may result in irreversible glycosylation of structural protein leading to myocardial stiffness [22,23], with impaired cardiac relaxation leading to diastolic and systolic dysfunction [24–26]. AGEs may also share in reactive oxygen species generation and inflammation through activation of AGE receptors [27], causing increased release of pro-inflammatory cytokines that contributes to augmentation of the adverse effects in the diabetic heart [28]. However, Linssen *et al.* [29] found that higher AGEs was associated with impaired diastolic and systolic LV function among only among nondiabetics and not observed among T2DM patients.

IGFBP-7 regulates insulin consumption and receptor activity by acting as a modulator for insulin-like growth factors [30]. It is a confirmed marker for diabetes and is associated with the severity of DD. In concordance with previous studies, our data revealed that IGFBP-7 was found to be significantly higher among DM-SD compared with DM-DD, DM-N patients and the two control groups; p < 0.001 and it was significantly higher among DM-DD compared with the two control groups; p < 0.001. Shaver *et al.* [16] found that level of IGFBP-7 was higher among DM-DD patients compared with the controls. Moreover, IGFBP-7 was identified as a HF biomarker in proteomic scans performed in a murine model of cardiac failure [31]. Among patients with chronic HF, elevated concentrations of IGFBP-7 predict major adverse cardiovascular events with impaired myocardial relaxation [8]. Guo *et al.* [32] found that IGFBP-7 has been implicated in fibrogenesis among DM, and was associated with increased collagen accumulation contributing to diastolic stiffness. Shaver *et al.* stated that IGFBP-7 played an important role in the early detection of DCM and cardiac fibrosis, enabling early intervention to attenuate disease progression [16].

Similar to several studies our results revealed that FBG, HbA1c and creatinine were significantly elevated among DM-SD and DM-DD compared with DM-N, and controls groups; p < 0.05 [16]. As expected, FBG and HbA1c were not elevated in the DD group because DD is not specific to DCM but may be due to the effect of hypertension or aortic stenosis [10]. The major abnormalities in DM are hyperglycemia, cardiac and systemic insulin resistance, which are included in the pathogenesis of DCM [6,33]. Stratton *et al.* [34] found a 1% reduction in HbA1c resulted in a 16% risk reduction in the development of HF, irrespective of other risk factors, such as obesity, hypertension, smoking or dyslipidemia. Moreover, among the newly diagnosed T2DM patients, the severity of DD was positively correlated with HbA1c levels [35]. Similar to the current study, Muhammad and Hashmi [36] reported an elevation in serum creatinine (>1.5) in DCM patients compared with diabetic patients without cardiomyopathy. However, further cohort studies are still needed among patients with DCM to assess the diagnostic and prognostic utility of serum biomarkers and their normal ranges to establish therapeutic strategies in order to prevent disease progression [37].

## Conclusion

Our study specified a panel of biomarkers to detect the diabetes-induced changes in cardiac structure and function existing at the early stage of DCM, and progression of DCM from subclinical diastolic dysfunction to overt HF. A panel of four biomarkers (TNF-α, Insulin, AGEs and creatinine) might be used for early detection of DCM (DM-DD) among T2DM patients with sensitivity of approximately 79% and specificity of 100%. A panel of two biomarkers (IL-6 and AGEs) were able to differentiate DM-SD from DM-DD with a sensitivity of 90.6%. A panel of three biomarkers (TNF-α, IL-6 and AGEs) can be used to discriminate between patients with DCM from DM with normal function with sensitivity and specificity of approximately 85%. These biomarkers can be used as predictors for early diagnosis of DCM, and may help in formulating strategic plans to slow or prevent the development of heart failure.

## Future perspective

The current study may aid in early diagnosis of DCM and help in formulating strategic plans to slow or prevent the development of HF. Further studies are needed to assess the validity of the studied biomarkers in a longitudinal prospective study as to achieve an early diagnosis of DCM in asymptomatic T2DM patients to prevent the irreversible fibrosis, leading to impaired contractility.

**Summary points**

- Diabetic cardiomyopathy (DCM) is a potential complication of diabetes. Diastolic dysfunction may be considered as the first functional abnormality in DCM and can be detected in 40–60% of asymptomatic diabetic patients using echocardiography.
- Detection of biomarkers that can enhance diagnosis of DCM before the occurrence of permanent complications is a promising approach.
- We assessed a panel of biomarkers (TNF-α, IL-6, IGFBP-7, AGEs, insulin and creatinine) alone or in combination with each other to predict early-stage of DCM.
- In conclusion, a panel of four biomarkers (TNF-α, insulin, AGEs and creatinine) might be used for early detection of diabetes mellitus-diastolic dysfunction among Type 2 diabetes mellitus patients with sensitivity of approximately 79% and specificity 100%. A panel of three biomarkers (TNF-α, IL-6 and AGEs) can be used to discriminate between patients with DCM from diabetes mellitus with normal function with sensitivity and specificity of approximately 85%.
